# *Streptococcus pneumoniae* Serotype 15A in Psychiatric Unit, Rhode Island, USA, 2010–2011

**DOI:** 10.3201/eid1811.120454

**Published:** 2012-11

**Authors:** Katherine Fleming-Dutra, Chukwuma Mbaeyi, Ruth Link-Gelles, Nicole Alexander, Alice Guh, Elizabeth Forbes, Bernard Beall, Jonas M. Winchell, Maria da Gloria Carvalho, Fabiana Pimenta, Maja Kodani, Cindy Vanner, Hilary Stevens, Diane Brady, Mardea Caulcrick-Grimes, Utpala Bandy, Matthew R. Moore

**Affiliations:** Centers for Disease Control and Prevention, Atlanta, Georgia, USA (K. Fleming-Dutra, C. Mbaeyi, R. Link-Gelles, A. Guh, B. Beall, J.M. Winchell, M.G. Carvalho, F. Pimenta, M. Kodani, H. Stevens, M.R. Moore);; Rhode Island Department of Health, Providence, Rhode Island, USA (N. Alexander, C. Vanner, D. Brady, M. Caulcrick-Grimes, U. Bandy);; Brown University–affiliated Hospitals, Providence (N. Alexander);; Brown University, Providence (E. Forbes)

**Keywords:** Streptococcus pneumoniae, pneumococcal infections, pneumonia, pneumococcal, serotype 15A, bacteria, antibiotic, antibacterial, disease outbreak, antimicrobial drugs

## Abstract

During a pneumococcal disease outbreak in a pediatric psychiatric unit in a hospital in Rhode Island, USA, 6 (30%) of 20 patients and staff were colonized with *Streptococcus pneumoniae* serotype 15A, which is not included in pneumococcal vaccines. The outbreak subsided after implementation of antimicrobial drug prophylaxis and enhanced infection control measures.

*Streptococcus pneumoniae*, or pneumococcus, causes an estimated 4 million illnesses in the United States annually (*1*). Pneumococcal disease outbreaks often occur in closed settings such as childcare facilities and hospitals. Control measures include vaccination, antimicrobial drug prophylaxis, and infection control (*2*). On January 26, 2011, the Rhode Island Department of Health was notified of 2 cases of invasive pneumococcal disease (IPD) and 2 cases of pneumonia that were associated with a unit (Unit 1) in a pediatric psychiatric hospital that had unusual infection control challenges. We investigated the outbreak to confirm the etiologic agent and prevent disease transmission.

## The Study

Children who have developmental disabilities and aggressive or self-injurious behavior are treated in Unit 1. Inpatient capacity is 17, and <5 outpatients are sometimes present during the day for group and individual therapy. The unit employs >100 staff members. Patients require constant supervision and intensive help with activities of daily living.

At the beginning of this investigation, we established outbreak case definitions. We defined confirmed pneumococcal disease as IPD (isolation of *S. pneumoniae* from a normally sterile site such as blood) or noninvasive (laboratory confirmation of *S. pneumoniae* from a nonsterile site in the setting of a compatible clinical illness, such as isolation from ear drainage samples from a patient with otitis media). We defined confirmed pneumonia as pneumonia diagnosed by a clinician, using chest radiographs confirmed as showing pneumonia by a radiologist. We defined suspected pneumonia as pneumonia diagnosed by a clinician with no radiologic studies obtained. Cases occurred in Unit 1 staff, patients, and visitors during the study period. We maintained active surveillance through May 1, but no cases occurred after February 23.

To identify risk factors for pneumococcal disease (*3*,*4*), we abstracted medical records of all Unit 1 patients (n = 30) during November 1, 2010–January 30, 2011. Unit 1 staff members completed questionnaires about respiratory illnesses experienced during December–February. The hospital informed families of then-current Unit 1 patients of the outbreak. Family members or visitors who reported illness were interviewed. All pertinent medical records were reviewed.

**Figure 1:**
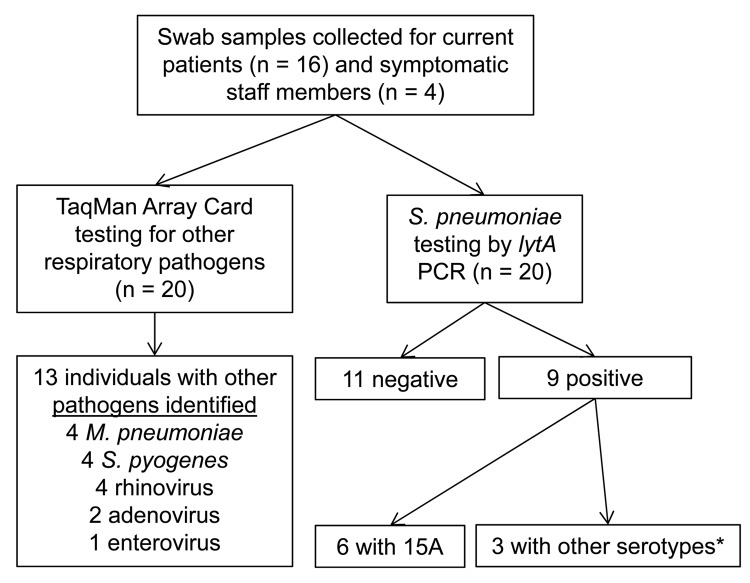
Respiratory pathogen carriage survey related to *Streptococcus pneumoniae* serotype 15A outbreak in a pediatric psychiatric hospital, Rhode Island, USA, December 25, 2010–January 31, 2011, performed on Unit 1 patients (n = 16) and symptomatic staff (n = 4) during January 29–February 2. No visitors were screened for respiratory pathogen carriage. Nasopharyngeal (NP) and oropharyngeal (OP) swab specimens were taken from each participant. TaqMan Array Card (TAC) used to test for influenza A (H1 and H3) and B, respiratory syncytial virus, human parainfluenza viruses 1–3, human metapneumovirus, rhinovirus, enterovirus, parechovirus, adenovirus, *Legionella* species, *Haemophilus influenzae, Streptococcus pyogenes, Mycoplasma pneumoniae, Chlamydophila pneumoniae,* and *Bordetella pertussis.* Asterisk indicates other pneumococcal serotypes identified in 3 persons, including 11A/D, 20, 34, and serotypes that were nontypeable by real-time PCR: 1, 2, 3, 4, 5, 6A/B/C, 6C, 7F/7A, 9V/9A, 11A/11D, 12F/(12A/44/46), 14, 15A/15F, 16F, 18/(18A/18B/18C/18F), 19A, 19F, 22F/22A, 23A, 23F, 33F/33A/37.

We collected available clinical specimens and conducted a survey to identify respiratory pathogens carried by patients and staff (Figure 1). We collected nasopharyngeal and oropharyngeal calcium alginate swab specimens from then-current Unit 1 patients (n = 16) and staff with ongoing respiratory symptoms (n = 4) during January 29–February 2. For pneumococcal carriage, swab specimens were processed as described (*5*,*6*). Three pneumococcal isolates were recovered (2 from blood, 1 from ear drainage samples). Swab specimens and isolates underwent specific real-time PCR that targeted the *lytA* gene (*6*), PCR-based serotyping (*7*,*8*), and multilocus sequence type determination (*9*). Antibacterial drug susceptibility testing was performed by using broth microdilution (*10*). Additional swabs stored in viral transport media were tested by solid-phase real-time PCR on TaqMan Array Cards (Life Technologies, Carlsbad, CA, USA), for 20 additional respiratory pathogen targets (*11*).

**Figure 2:**
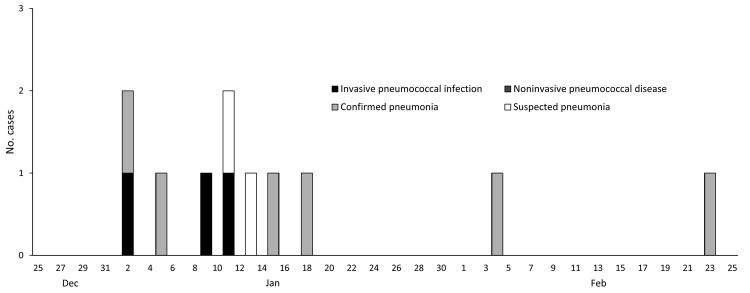
Epidemic curve of *Streptococcus pneumoniae* outbreak in a pediatric psychiatric hospital, Rhode Island, USA, December 25, 2010–January 31, 2011, for invasive pneumococcal disease, noninvasive pneumococcal disease, confirmed pneumonia, and suspected pneumonia. *Staff members; †family members.

Twenty patients resided on Unit 1 during December 25, 2010–January 31, 2011 (Table 1). Among Unit 1 patients, staff, and visitors, the following cases were identified: 3 confirmed pneumococcal disease, 6 confirmed pneumonia, and 2 suspected pneumonia (Figure 2). Three case-patients were hospitalized (2 with IPD, 1 with confirmed pneumonia). Among the 20 patients, the cases of 5 (attack rate 25%) met an outbreak case definition (Table 2). In addition, 1 staff member had IPD, 1 had confirmed pneumonia, and 1 had suspected pneumonia (Table 2, attack rate <3%). These staff members provided direct care to all Unit 1 patients. Three visitors had confirmed pneumonia. Adults (3 staff and 3 visitors) who became ill during the outbreak and for whom ages were available (5 of 6) ranged in age from 27 to 56 years.

**Table 1:** Characteristics of 20 psychiatric unit patients during *Streptococcus pneumoniae* serotype 15A outbreak, Rhode Island, USA, December 25, 2010–January 31, 2011

Characteristic	No. (%) patients
Male sex	14 (70.0)
Median age, y (range)	13 (4–24)
Race	
White	16 (80.0)
Black	4 (20.0)
Other/unknown	0
Underlying medical conditions	
Asthma*	6 (30.0)
Chronic heart disease	2 (10.0)
Other risk factors for pneumococcal infection†	0
Median length of stay, days on Unit 1 as of January 30, 2011 (range)	71 (1–706)
Receiving systemic antimicrobial drugs during January for illness unrelated to outbreak	1 (5.0)
No. patients on Unit 1 as of January 30, 2011	
Current in-patient	15 (75.0)
Day program patient‡	1 (20.0)
Discharged (since December 25, 2010)	4 (5.0)

*None were treated with systemic steroids (i.e., none had an indication for vaccine) (*3*,*4***).** **†Includes chronic lung disease (other than asthma), diabetes mellitus, cerebrospinal fluid leaks, cochlear implant, systemic steroid use, sickle cell disease, congenital or acquired asplenia, HIV infection, chronic renal failure, nephritic syndrome or kidney disease with dialysis, bone-marrow or organ transplant, diseases associated with treatment with immunosuppressive drugs or radiation therapy, including malignant neoplasms, leukemia, lymphoma, and Hodgkin disease (***3*,*4***).** ‡Day program patients were treated in Unit 1 during the day and returned home to stay overnight with their families.

**Table 2:** Cases, clinical isolates, and results of pneumococcal carriage and respiratory pathogen survey associated with *Streptococcus pneumoniae* serotype 15A outbreak, Rhode Island, USA, December 25, 2010–January 31, 2011*

Case status	Case no.	Onset date	Culture date, specimen and result	Chest imaging results	Treatment	Outcome	Carriage	Sero	Other pathogens by TAC†
IPD	S1	Jan 9	Jan 14, blood, *S. pneumoniae* 15A	Right upper lobe pneumonia	Moxifloxacin	Rec	Neg		Neg
IPD	P1	Jan 11	Jan 25, blood, *S. pneumoniae* 15A	Negative	Ceftriaxone	Rec	Pos	15A	Rhinovirus, adenovirus
Noninvasive pneumococcal disease	P2	Jan 2	Jan 28, otorrhea, *S. pneumoniae* 15A		Cefuroxime, topical ofloxacin	Rec	Pos	15A	*Mycoplasma pneumoniae*
Confirmed pneumonia	P3	Jan 2		Left lower lobe pneumonia	Amoxicillin/ clavulanate, azithromycin	Rec	Pos	NT‡	Rhinovirus
Confirmed pneumonia	S2	Jan 5		Left upper lobe pneumonia	Amoxicillin/ clavuanate, ciprofloxacin	Rec	NS		NS
Confirmed pneumonia	V1	Jan 15		Right basilar pneumonia	Moxifloxacin	Rec	NS		NS
Confirmed pneumonia	P4	Jan 18		Right lower lobe pneumonia	Ceftriaxone, ampicillin, amoxicillin	Rec	Neg		*M. pneumoniae*
Confirmed pneumonia	V2	Feb 4		Left lower lobe pneumonia	Doxycycline, levofloxacin	Rec	NS		NS
Confirmed pneumonia§	V3	Feb 23		Left lower lobe pneumonia	Unk	Unk	NS		NS
Suspected pneumonia	S3	Jan 11			Azithromycin	Rec	NS		NS
Suspected pneumonia	P5	Jan 13			Clindamycin	Rec	Neg		Neg
Non-case	P6						Neg		*S. pyogenes*
Non-case	P7						Neg		Adenovirus
Non-case	P8						Neg		Neg
Non-case	P9						Pos	34	*S. pyogenes*
Non-case	P10						Neg		Neg
Non-case	P11						Pos	15A	Enterovirus
Non-case	P12						Pos	15A	Neg
Non-case	P13						Pos	11 A/D, 20	*S. pyogenes*
Non-case	P14						Neg		Neg
Non-case	P15						Pos	15A	Rhinovirus, *M. pneumoniae*
Non-case	P16						Pos	15A	*M. pneumoniae*
Non-case	S4						Neg		Rhinovirus
Non-case	S5						Neg		Neg
Non-case	S6						Neg		*S. pyogenes*

*Sero, serotype; TAC, TaqMan Array Card; IPD, invasive pneumococcal disease; S, staff; rec, recovered; neg, negative; P, patient; pos, positive; NS, no carriage screen; V, visitor; NT, nontypeable; Unk, unknown. **†TAC used to test for influenza viruses A (H1 and H3) and B, respiratory syncytial virus, human parainfluenza viruses 1–3, human metapneumovirus, rhinovirus, enterovirus, parechovirus, adenovirus, *Legionella* species, *Haemophilus influenzae*, *Streptococcus pyogenes*, *Mycoplasma pneumoniae*, *Chlamydophila pneumoniae*, and *Bordetella pertussis*.** **‡NT by real-time PCR (for serotypes: 1, 2, 3, 4, 5, 6A/B/C, 6C, 7F/7A, 9V/9A, 11A/11D, 12F/(12A/44/46), 14, 15A/15F, 16F, 18/(18A/18B/18C/18F), 19A, 19F, 22F/22A, 23A, 23F, 33F/33A/37).** §Refused further involvement with investigation.

All 3 clinical isolates were identified as *S. pneumoniae* serotype 15A, sequence type 63, (Table 2) with matching antimicrobial drug susceptibility patterns. Nine (45%) of 20 persons tested carried pneumococcus; of those, 6 (30%) carried serotype 15A. Rhinovirus, *Streptococcus pyogenes,* and *Mycoplasma pneumoniae* were additional pathogens most frequently identified by using TaqMan Array Cards (Figure 1, Table 2).

We assessed Unit 1 infection control practices; in particular, for staff compliance with hand and respiratory hygiene. The hand hygiene audit revealed that staff members had performed hand hygiene in 4 (24%) of 17 instances before patient contact and 11 (79%) of 14 times after patient contact, for an overall compliance of 15 (48%) of 31 opportunities. In addition,, supplies (e.g., gloves) were kept in locked cabinets because of safety concerns. Staff reported that patients, because of their developmental delays, were often unable to appropriately manage their respiratory secretions.

The presence of 6 carriers of serotype 15A on Unit 1 during the carriage survey indicated the potential for continued transmission and disease. The hospital mandated hand and respiratory hygiene training for all Unit 1 staff, administered during February 4–8. We recommended high-dose amoxicillin prophylaxis (90 mg/kg/day divided into 2 doses, maximum of 1,000 mg, for 5 days) for all Unit 1 patients, which began on February 7. After control measures were fully implemented, 1 case of pneumonia was confirmed in a patient’s parent on February 23, but the etiology was not identified. No additional cases occurred among Unit 1 patients and staff members during the subsequent 3 months.

## Conclusions

Laboratory and epidemiologic evidence indicates that an outbreak of *S. pneumoniae* serotype 15A infection occurred on Unit 1. Increased transmission opportunities likely resulted from infection control challenges in the unit. Because serotype 15A is not included in any current pneumococcal vaccine, we used antimicrobial drug prophylaxis as an immediate intervention to reduce transmission. However, infection control was the critical long-term control measure. We modified acute-care hospital infection control practices for the unique environment of Unit 1, such as scheduling hand hygiene sessions every 2–3 hours for all patients, rather than expecting patients to understand when to perform hand hygiene. Furthermore, other respiratory pathogens were present on Unit 1, which could have contributed to disease and facilitated pneumococcal transmission by increasing nasopharyngeal colonization (*12*). The same infection control lapses that led to the *S. pneumoniae* serotype 15A outbreak likely led to transmission of these respiratory pathogens.

Since the introduction of the 7-valent pneumococcal conjugate vaccine, nasopharyngeal carriage of serotype 15A has increased (*13*). Among Massachusetts children 3 months–7 years of age, serotype 15A carriage prevalence was 4% in 2007 (*14*), compared with 30% in this outbreak. Since the introduction of the 7-valent pneumococcal conjugate vaccine, serotype15A sequence type 63 has also caused an increasing proportion of penicillin-nonsusceptible IPD in the United States (*15*). The sequence type and antimicrobial drug susceptibilities of the serotype 15A strain from this outbreak are characteristic of a single, internationally disseminated serotype 15A strain (*15*). No other increases in IPD or serotype 15A were detected by the Rhode Island Department of Health during this period, indicating that the outbreak was restricted to Unit 1.

This investigation had several limitations. First, diagnostic tests were performed at the discretion of treating clinicians, and specific diagnostic testing was not performed for most pneumonia case-patients. In addition, we used TaqMan Array Cards <3 weeks after symptom onset, which decreased our ability to detect all bacterial and viral co-factors that possibly contributed to this outbreak. Because of the small size of the patient cohort, statistical tests would not have power to assess risk factors for pneumococcal disease or carriage. Finally, we did not assess the knowledge of hand hygiene among staff after the training to evaluate its effectiveness.

In conclusion, this outbreak was associated with *S. pneumoniae* serotype 15A, a serotype not included in available pneumococcal vaccines. The outbreak subsided after patients received antimicrobial drug prophylaxis. The hospital instituted a hand hygiene monitoring program in response to our recommendations and enhanced infection control practices were implemented, especially careful adherence to hand and respiratory hygiene. Hand and respiratory hygiene training and monitoring are critical for infection control in units that serve patients with special cognitive needs.
